# Deep consistency-preserving hash auto-encoders for neuroimage cross-modal retrieval

**DOI:** 10.1038/s41598-023-29320-6

**Published:** 2023-02-09

**Authors:** Xinyu Wang, Xianhua Zeng

**Affiliations:** College of Computer Science and Technology, Chongqing University of Posts and Telecommunications, Chongqing, 400065 China

**Keywords:** Engineering, Computational models, Image processing, Machine learning

## Abstract

Cross-modal hashing is an efficient method to embed high-dimensional heterogeneous modal feature descriptors into a consistency-preserving Hamming space with low-dimensional. Most existing cross-modal hashing methods have been able to bridge the heterogeneous modality gap, but there are still two challenges resulting in limited retrieval accuracy: (1) ignoring the continuous similarity of samples on manifold; (2) lack of discriminability of hash codes with the same semantics. To cope with these problems, we propose a Deep Consistency-Preserving Hash Auto-encoders model, called DCPHA, based on the multi-manifold property of the feature distribution. Specifically, DCPHA consists of a pair of asymmetric auto-encoders and two semantics-preserving attention branches working in the encoding and decoding stages, respectively. When the number of input medical image modalities is greater than 2, the encoder is a multiple pseudo-Siamese network designed to extract specific modality features of different medical image modalities. In addition, we define the continuous similarity of heterogeneous and homogeneous samples on Riemann manifold from the perspective of multiple sub-manifolds, respectively, and the two constraints, i.e., multi-semantic consistency and multi-manifold similarity-preserving, are embedded in the learning of hash codes to obtain high-quality hash codes with consistency-preserving. The extensive experiments show that the proposed DCPHA has the most stable and state-of-the-art performance. We make code and models publicly available: https://github.com/Socrates023/DCPHA.

## Introduction

Recently, various advanced medical imaging technologies have been applied in modern clinical analysis with the advancement of medical care^1^. Hospitals are generating a large number of multi-modal neuroimages every moment, therefore, it is necessary to establish an effective neuroimage cross-modal approximate nearest neighbor retrieval system to assist clinicians in navigating the data. Neuroimage cross-modal retrieval aims to provide doctors with similar neuroimages from different modalities that have been diagnosed. An effective neuroimage cross-modal retrieval system can reduce the error rate of clinical diagnosis for novice doctors and improve the efficiency of clinical diagnosis for skilled physicians.

The remarkable achievements have been made in large-scale data processing based on deep neural network in computer vision^2–5^, Internet of Things (IoT)^6–8^, nearest neighbor retrieval^9, 10^, and intelligent networks^11, 12^. The nearest neighbor retrieval methods are solved by learning discriminative representations in the common space, which can be roughly classified into cross-modal hash retrieval and cross-modal real-value retrieval by classifying the types of values in the common space^10, 13^. Cross-modal hashing is an efficient method to embed high-dimensional heterogeneous modal feature descriptors into a low-dimensional Hamming space. Due to the trade-off between retrieval efficiency and storage cost, learning to hash has been widely used in approximate nearest neighbor retrieval of large-scale multi-media data, in particular, using cross-modal hashing to assist doctors in effective clinical diagnosis has also attracted increasing attention from researchers.

Since features of different modalities usually belong to various data distributions and are generated from different manifold spaces. Therefore, a basic challenge of cross-modal retrieval is to bridge the modality gap. Most existing cross-modal hashing methods have been available to bridge the heterogeneous modality-gap^14–16^, but there are still two challenges leading to the limitation of retrieval accuracy: (1) ignoring the continuous similarity of samples on stream shape; (2) lack of discriminability of hash codes with the same semantics. Our research argued that (1) is the reason for (2) and (2) is the result of (1). Therefore, we propose a Deep Consistency-Preserving Hash Auto-encoders model, called DCPHA, based on the multi-manifold property of multi-modal hash codes distributed in Hamming space. In addition, we define the continuous similarity of heterogeneous and homogeneous samples on Riemann manifolds from the perspective of multiple sub-manifolds, respectively, and propose two constraints, i.e., multi-semantic consistency and multi-manifold similarity-preserving. And we prove theoretically that the multi-manifold similarity-preserving constraint has manifold preserving invariance.

The main contributions of our work can be summarized as follows:

1. We propose a Deep Consistency-Preserving Hash Auto-encoders model, called DCPHA, based on the multi-manifold property of the feature distribution for neuroimage cross-modal retrieval. DCPHA is an end-to-end model consisting of asymmetric auto-encoders and two semantics-preserving attention branches.
2. We propose multi-semantic consistency and multi-manifold similarity-preserving constraints based on the multi-manifold property of multi-modal hash codes. And it is proved theoretically that the multi-manifold similarity-preserving constraint has manifold preserving invariance.
3. Without loss of generality, we comprehensively evaluate the DCPHA on four benchmark datasets and implement detailed ablation experiments to validate the effectiveness of the DCPHA. The extensive experiments demonstrate the advantages of the proposed DCPHA compared to 15 advanced cross-modal retrieval methods.

## Deep consistency-preserving hash auto-encoders

**Figure 1:**
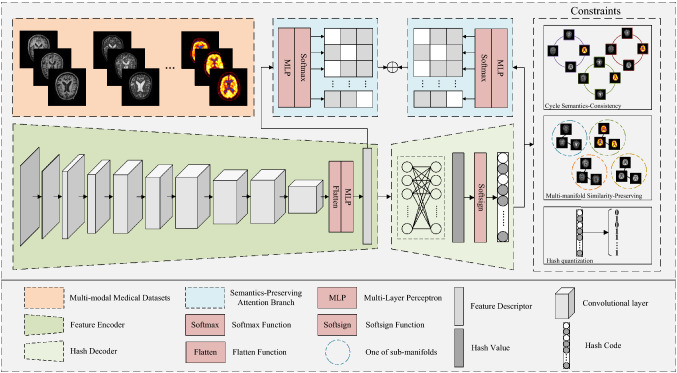
The proposed DCPHA model consists of an asymmetric auto-encoders and two semantics-preserving attention branches. The encoder is used to extract features from neuroimages of different modalities, and the decoder is designed to map the features into Hamming space by a non-linear transformation. Best view in color.

In this section, the proposed model DCPHA is described in detail, including formulations, deep architecture and objective function. The deep architecture of DCPHA is shown in Fig. 1. The DCPHA model consists of asymmetric auto-encoders and two semantics-preserving attention branches. The encoder is used to extract features from neuroimages of different modalities, and the decoder is designed to map the features into Hamming space by a non-linear transformation. The semantics-preserving attention branches work in the encoding and decoding stages respectively to ensure that both the learned features and the hash codes have semantics-consistency. And two constraints, i.e., multi-semantic consistency and multi-manifold similarity-preserving, are embedded in the learning of hash codes to obtain high-quality hash codes with discriminative.

### Notations and definitions

In this subsection, the notations and definitions mentioned in the following equations are introduced. Without loss of generality, we suppose that there are ${\mathscr {N}}$ multi-modal sample sets in the sample space $\psi$, $\psi =\left\{ X_i\right\} , i\in \left[ 1,{\mathscr {N}}\right]$. Each of multi-modal sample sets $X_i$ consists of different medical scan imagings from the same subject (e.g. MRI and PET), $X_i=\left\{ x_i^m\right\} , m\in \left[ 1,{\mathscr {M}}\right]$, where ${\mathscr {M}}$ denotes the number of different medical scan imagings. $x_i^m$ denotes the *i*-th subject of the *m*-th modality, assuming dimension ${\mathscr {Z}}$. Since the samples within the same multi-modal sample set originate from the same subject, they naturally share the same semantic, which is the reason why our method is appropriate for neuroimages. A one-hot vector $\ell _i$ is assigned to each multi-modal sample set, $\ell _i=\left[ l_1,l_2,\cdots ,l_c,\cdots ,l_C\right]$, where *C* denotes the number of categories. When the multi-modal sample set $X_i$ belongs to the *c*-th category, $l_c=1$ and the rest is 0. DCPHA consists of an asymmetric encoder and decoder. The purpose of the encoder is to learn the features $f_i^m$ of sample $x_i^m$, assuming that the dimension of $f_i^m$ is ${\mathscr {D}}$, where ${\mathscr {D}}\ll {\mathscr {Z}}$. The decoder is designed to map the features $f_i^m$ into Hamming space by a non-linear transformation. Let the hash code of sample $x_i^m$ is $h_i^m$, $h_i^m\in \left\{ -1,1\right\} ^{\mathscr {K}}$, and our goal aims to learn an end-to-end non-linear hash function ${\mathscr {F}}$ to extract features of multi-modal medical imaging and encode them into high-quality hash codes with semantics-consistency and similarity-preserving, $h_i^m={\mathscr {F}}\left( x_i^m;\theta \right)$. The terms, notations, definitions and types involved in this work are comprehensively shown in Table 1.

**Table 1:** The terms, notations, definitions and types involved in this work.

Notation	Definition	Type	Shape
${\mathscr {M}}$	The number of different medical scan imagings	Constant	/
${\mathscr {N}}$	The number of multi-modal sample sets	Constant	/
*C*	The number of categories	Constant	/
$\psi$	Medical multi-modal sample space	Array	$({\mathscr {N}},{\mathscr {M}},{\mathscr {Z}})$
$X_i$	Multi-modal sample set	Matrix	$({\mathscr {M}},{\mathscr {Z}})$
$x_i^m$	Multi-modal sample	vector	$(,{\mathscr {Z}})$
$f_i^m$	The vectorized features corresponding to the multi-modal sample $x_i^m$	vector	$(,{\mathscr {D}})$
$h_i^m$	The hash code corresponding to the multi-modal sample $x_i^m$	vector	$(,{\mathscr {K}})$
$\ell _i$	The semantic label corresponding to the multi-modal sample set $X_i$	vector	(, *C*)

**Figure 2:**
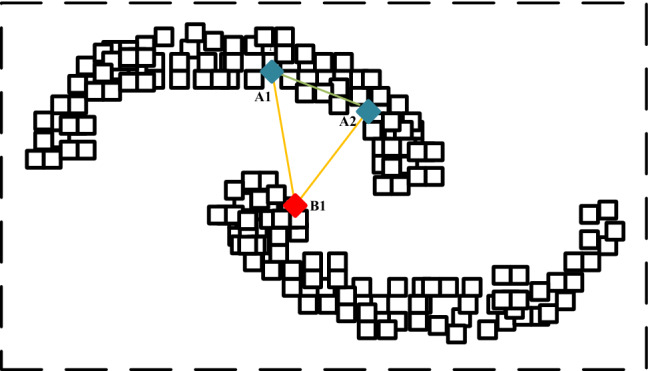
The visualization of multiple sub-manifolds in local sample space. Points A1, A2 and B1 are from two different manifolds. The green solid line connects two homogeneous manifold samples, i.e. the similarity between two homogeneous manifold samples, and the yellow solid line links two heterogeneous manifold samples, i.e. the similarity between two heterogeneous manifold samples. Best view in color.

The previous works^17–20^ has illustrated that multi-modal data contain multiple sub-manifolds. The visualization of multiple sub-manifolds in local sample space is shown in Fig. 2. We define the sub-manifold similarity and multi-manifold similarity from local and global respectively, as follows.

#### Definition 1

**Heterogeneous manifold similarity**. A local manifold similarity calculation definition. Assuming that there are ${\mathscr {M}}$ modal neuroimages in the sample space, and each modality contains ${\mathscr {N}}$ samples, then the heterogeneous manifold similarity ${{\textbf {S}}}_{{\textbf {H}}}$ is defined for any two samples of different modalities as Eq. (1):

1 $$\begin{aligned} {{\textbf {S}}}_{{\textbf {H}}} = \begin{bmatrix} S_H(h_1^m,h_1^n) &{} \cdots &{} S_H(h_1^m,h_{\mathscr {N}}^n) \\ \vdots &{} \ddots &{} \vdots \\ S_H(h_{\mathscr {N}}^m,h_1^n) &{} \cdots &{} S_H(h_{\mathscr {N}}^m,h_{\mathscr {N}}^n) \\ \end{bmatrix}_{{\mathscr {N}}\times {\mathscr {N}}} \end{aligned}$$

with

2 $$\begin{aligned} S_H\left( h_i^m,h_j^n\right)= & {} e^\frac{-D^2\left( h_i^m,h_j^n\right) }{\tau } \end{aligned}$$

3 $$\begin{aligned} D\left( h_i^m,h_j^n\right)= & {} \left\{ \begin{array}{ll} \sqrt{1-e^{-d\left( h_i^m,h_j^n\right) }}, &{} l_i=l_j \\ \sqrt{e^{-d\left( h_i^m,h_j^n\right) }}, &{} l_i\ne l_j \end{array} \right. \end{aligned}$$

where $S_H\left( h_i^m,h_j^n\right)$ denotes the similarity of the heterogeneous manifold between the *i*-th sample of the *m*-th modal and the *j*-th sample of the *n*-th modal and the calculation method is shown in Eq. (2). $\tau$ is the heat kernel constant. $D(\cdot )$ in Eq. (3) is the modified distance metric based on the standard euclidean distance $d(\cdot )$.

#### Definition 2

**Homogeneous manifold similarity**. A local manifold similarity calculation definition. In the sample space, the homogeneous manifold similarity ${{\textbf {S}}}_{{\textbf {I}}}$ between samples from the same modality is defined as Eq. (4):

4 $$\begin{aligned} {{\textbf {S}}}_{{\textbf {I}}} = \begin{bmatrix} S_I(h_1^\cdot ,h_1^\cdot ) &{} \cdots &{} S_I(h_1^\cdot ,h_{\mathscr {N}}^\cdot ) \\ \vdots &{} \ddots &{} \vdots \\ S_I(h_{\mathscr {N}}^\cdot ,h_1^\cdot ) &{} \cdots &{} S_I(h_{\mathscr {N}}^\cdot ,h_{\mathscr {N}}^\cdot ) \\ \end{bmatrix}_{{\mathscr {N}}\times {\mathscr {N}}} \end{aligned}$$

with

5 $$\begin{aligned} S_I(h_i^\cdot ,h_j^\cdot )=\frac{{h_i^{\cdot }}^T \cdot h_j^\cdot }{\Vert h_i^{\cdot }\Vert \Vert h_j^{\cdot }\Vert } \end{aligned}$$

where $S_I(h_i^\cdot ,h_j^\cdot )$ denotes the homogeneous manifold similarity between the *i*-th sample and the *j*-th sample from the same modal. The calculation method is the dot product between $\ell _2$ normalized $h_i^{\cdot }$ and $h_j^{\cdot }$ (i.e. cosine similarity) as shown in Eq. (5).

#### Definition 3

**Multi-manifold similarity**. A global manifold similarity calculation definition. Assuming that there are ${\mathscr {M}}$ modal neuroimages in the sample space and each modality contains ${\mathscr {N}}$ samples, then the multi-manifold similarity ${{\textbf {S}}}_{{\textbf {M}}}$ is defined as Eq. (6):

6 $$\begin{aligned} {{\textbf {S}}}_{{\textbf {M}}} = \begin{bmatrix} S_I^1 &{} \cdots &{} S_H^{1,{\mathscr {M}}} \\ \vdots &{} \ddots &{} \vdots \\ S_H^{{\mathscr {M}},1} &{} \cdots &{} S_I^{\mathscr {M}} \\ \end{bmatrix}_{{\mathscr {M}}{\mathscr {N}}\times {\mathscr {M}}{\mathscr {N}}} \end{aligned}$$

where $S_I^1$ denotes the homogeneous manifold similarity between the samples from the *1*-th modality, and $S_I^{{\mathscr {M}}}$ similarly. $s_H^{1,{\mathscr {M}}}$ denotes the heterogeneous manifold similarity between the *1*-th modal sample and the ${\mathscr {M}}$-th modal sample, and $S_H^{{\mathscr {M}},1}$ similarly.

### Objective functions and theory

In this subsection, the theoretical derivation of the proposed multi-semantic consistency and multi-manifold similarity-preserving constraints is presented. Alexey et al.^21^ propose that the criterion for a good feature representation should ensure that the mapping from the input image $x_i^m$ to the feature $f_i^m$ should satisfy two requirements: (1) There must be at least one feature that is similar for images of the same semantics. (2) there must be at least one feature that is sufficiently different for images of different semantics. However, the previous works^14–16^ can over-satisfy both requirements for hash codes, because these works ignore the fact that samples with the same semantics have contiguous similarity on manifold. Constructing the similarity matrix directly using semantic labels leads to samples with the same semantics being encoded into the same hash code, causing the lack of discriminability between hash codes with the same semantics. To solve the problem, we propose a multi-semantic consistency loss and a multi-manifold similarity-presering loss. The multi-semantic consistency ensures that hash codes with different semantics are discriminative. On this basis, multi-manifold similarity-preserving defines continuous similarity among samples in terms of multiple sub-manifolds, ensuring that hash codes with the same semantics have discriminability as well.

#### Multi-semantic consistency

The multi-semantic consistency constraint is to align the intermediate features generated by the encoder and the hash codes generated by the decoder with the high-level semantics of the input samples to guarantee that the final generated hash codes with different semantics have case-level discriminability, which is calculated as follows.

First, the sample $x_i^m$ is learned by encoder to feature $f_i^m$, $f_i^m=Decoder\left( x_i^m\right)$, and the feature $f_i^m$ is passed through the Semantic Preserving Attention Branch (SPAB) to obtain the feature prediction classification label $y_i^m$, $y_i^m=SPAB_E(f_i^m)$. The decoder is designed to map the features into Hamming space by a non-linear transformation. The $f_i^m$ is fed into the decoder to obtain the hash code $h_i^m$, $h_i^m=Decoder\left( f_i^m\right)$. The hash code $h_i^m$ is input into the SPAB which works in the decoding stage to obtain the hash code prediction classification label $r_i^m$, $r_i^m=SPAB_D(h_i^m)$. The multi-semantic consistency loss is shown in Eq. (7), where $\parallel \cdot \parallel _{\mathscr {F}}$ denotes the *Frobenius* *normalized*.

7 $$\begin{aligned} {\mathscr {J}}_1=\sum _{m=1}^{\mathscr {M}}\sum _{i=1}^{\mathscr {N}} \parallel y_i^m-\ell _i\parallel _{\mathscr {F}}+ \parallel r_i^m-\ell _i\parallel _{\mathscr {F}}+ \parallel y_i^m-r_i^m\parallel _{\mathscr {F}} \end{aligned}$$

#### Multi-manifold similarity-preserving

With the basis of multi-semantic consistency, multi-manifold similarity-preserving defines continuous similarity among samples in terms of multiple sub-manifolds, ensuring that hash codes with the same semantics have discriminability as well. According to previous work^18–20^, in the sample space, neuroimages of different modalities are distributed in different sub-manifolds. They are aggregated into a sophisticated multi-manifold structure. Based on the statements of Definition. (1)(2)(3), we derive the following optimization equation as Eq. (8):

8 $$\begin{aligned} {\mathscr {J}}_2=\sum _{m,n=1}^{M}\sum _{i,j=1}^{N}\left( \log \left( 1+e^{S_M\left( h_i^m, h_j^n\right) }\right) -I\left( \ell _i, \ell _j\right) \times S_M\left( h_i^m, h_j^n\right) \right) \end{aligned}$$

where $S_M\left( \cdot ,\cdot \right)$ denotes the multi-manifold similarity. $I\left( \cdot ,\cdot \right)$ is an indicator function that has a value of 1 if $\ell _i=\ell _j$ and 0 otherwise. Other notations and the corresponding explanations can be found in Table 1. ${\mathscr {J}}_2$ is the similarity-preserving loss which is defined on multi-manifolds, allowing samples with the same semantics are decoded into hash codes with discriminative.

Belkin^22^ used the correspondence between the Laplace and the Laplace-Beltrami operator on manifold, and the connections to heat equation, and proposed a non-linear dimensionality reduction method from Riemann space to Euclidean space (i.e. Laplacian Eigenmaps). The objective function as follows Eq. (9):

9 $$\begin{aligned} {\mathscr {L}}_{laplacican}=\sum _{m,n=1}^{M}\sum _{i,j=1}^{N}\frac{1}{2}S_{i,j}\times \parallel H_i^m-H_j^n\parallel ^2_2 \end{aligned}$$

where $H_i^m=\frac{h_i^m}{\parallel h_i^m\parallel }$, i.e. standardized feature vector.

##### Theorem 1

Subject to $log\left( 1+e^{S_M\left( h_i^m,h_j^n\right) }\right) =2S_M\left( h_i^m,h_j^n\right)$, then ${\mathscr {J}}_2$ is equivalent to ${\mathscr {L}}_{laplacican}$, i.e. ${\mathscr {J}}_2$ has manifold preserving invariance.

The procedure of the theoretical proof of Theorem 1 is placed in the supplementary material. It indicates that minimizing Eq. (8) is a standard manifold embedding problem formulated by equivalent to Eq. (9). Multi-manifold similarty-preserving term essentially provides a measure of sub-manifold similarity-preserving. Therefore, Eq. (8) can be a reasonable explanation for multi-manifold similarity-preserving.

Combining Eqs. (7)(8), the objective function of DCPHA is:

10 $$\begin{aligned} {\mathscr {J}}={\alpha {\mathscr {J}}}_1+{\beta {\mathscr {J}}}_2+\sum _{m,n=1}^M\sum _{i,j=1}^N \parallel h_i^m-{{\textbf {1}}}\parallel _1+\parallel h_j^n-{{\textbf {1}}}\parallel _1 \end{aligned}$$

where $\alpha$ and $\beta$ are the contribution weight parameters of ${\mathscr {J}}_1$ and ${\mathscr {J}}_2$, respectively. The third is a regularization term, which is used to avoid gradient vanishing^23^.

### Refinement learning and optimization

The network structure of DCPHA consists of asymmetric auto-encoders and two semantics-preserving attention branchinges which working in the feature encoding and hash decoding stages, respectively. The encoder adopts a standard CNN network structure. The decoder uses a light-weight fully-connected networks. The semantics-preserving attention branch is a linear multi-layer perceptron model. Therefore Eq. (10) is a non-convex function with multiple parameters. We used a stochastic gradient descent method and iterative learning strategy with Adam optimizer^24^ to learn the parameters and update the network. The complete training of DCPHA consists of three steps: (1) Pre-training encoder, (2) Pre-training decoder and (3) Fine-tuning DCPHA, which are described in detail below.
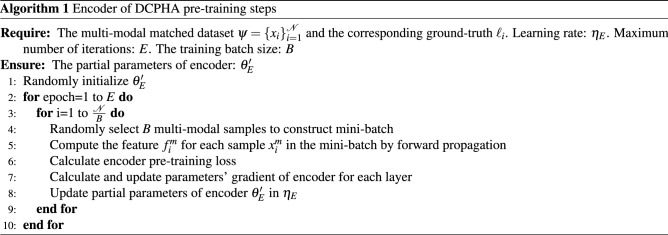

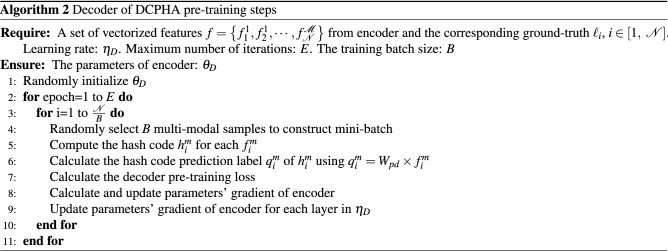

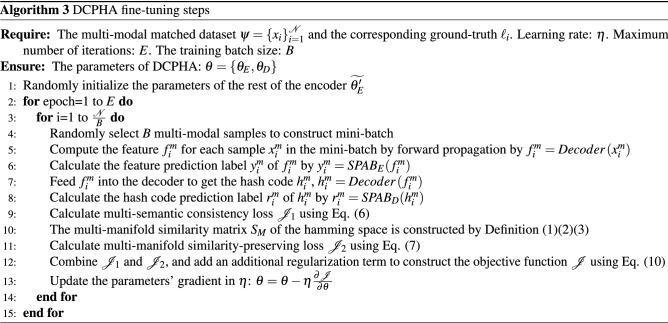


## Experiment

### Implementation details

All experiments were conducted on a Tesla V100-SXM2 GPU using same setting. To ensure impartiality and objectivity, all comparison models adopt AlexNet as the backbone network for feature extraction. All comparison models, except that the backbone network adopts the same configuration, are all original code implementations. The batchsize is 20 and the iterations is 500. The initial learning rate is set to $10^{-6}$.

### Datasets

**ADNI2**^25^ contained 579 subjects with T1-weighted sMRI and 500 subjects with PET. we adopt a single slice and strong pairing data preprocessing method. Finally, we collected 300 pairs (600 images) of sMRI and PET neuroimages as datasets.

**OASIS3**^26^. We collected MRI T1-weighted and PET images of 300 subjects from the OASIS3 dataset, with a total of 600 images as the dataset. We strongly matched two different modal images of the same subject to form a cross-modal paired dataset for training. We divided the above datasets into training-set and test-set in the ratio of 8/2. The datasets generated and analysed during the current study are available from the corresponding author on reasonable request.

### Compare with 15 advanced methods

In this experiments, We used the mean average precision (mAP) scores of all returned results with cosine similarity as a quantitative metric. The mAP scores jointly consider ranking information and precision and are widely used performance evaluation criteria in cross-modal hash. We report the mAP scores of the compared methods for two different cross-modal retrieval tasks: (1) retrieving PET samples using T1-wighted MRI queries (M$\rightarrow$P) and (2) retrieving T1-wighted MRI samples using PET queries (P$\rightarrow$M). On the premise of objectivity and impartiality, the comparison experiments on ADNI2 and OASIS3 datasets are shown in Tables 2, 3, respectively. From the results, DCPHA achieves state-of-the-art performance on the test-set of each dataset. The detailed analysis is as follows.

**Table 2:** The mAP scores of cross-modal retrieval on ADNI2 with different lengths of hash codes.

Method	16 bits			32 bits			64 bits			128 bits		
	M$\rightarrow$P	P$\rightarrow$M	Aver	M$\rightarrow$P	P$\rightarrow$M	Aver	M$\rightarrow$P	P$\rightarrow$M	Aver	M$\rightarrow$P	P$\rightarrow$M	Aver
DHN^27^	0.5853	0.5864	0.5858	0.5176	0.5461	0.5318	0.5339	0.5318	0.5328	0.5397	0.5433	0.5415
DSH^28^	0.6236	0.6250	0.6243	0.5883	0.5929	0.5906	0.5615	0.5675	0.5645	0.5777	0.5950	0.5864
DPSH^29^	0.5984	0.5773	0.5878	0.5515	0.5736	0.5625	0.5780	0.5844	0.5812	0.5802	0.5765	0.5783
DAPH^30^	0.5117	0.5292	0.5205	0.5587	0.5683	0.5635	0.5178	0.5190	0.5184	0.5244	0.5240	0.5242
HashNet^31^	0.5341	0.5724	0.5533	0.5575	0.5735	0.5655	0.5718	0.5784	0.5751	0.5470	0.5558	0.5514
DSDH^32^	0.5649	0.5703	0.5676	0.5681	0.5810	0.5745	0.5523	0.5432	0.5477	0.5890	0.5734	0.5812
LCDSH^33^	0.5697	0.5717	0.5707	0.5616	0.5861	0.5738	0.5826	0.5570	0.5698	0.5635	0.5559	0.5597
ADSH^34^	0.5932	0.5986	0.5959	0.5789	0.6016	0.5902	0.6045	0.6391	0.6218	0.5852	0.6213	0.6032
DIHN^35^	0.5598	0.5978	0.5788	0.6015	0.5924	0.5969	0.6002	0.6371	0.6187	0.6076	0.6179	0.6127
DSCMR^36^	0.4745	0.4742	0.4744	0.4244	0.4297	0.4271	0.4334	0.4264	0.4299	0.4140	0.4139	0.4139
IDHN^37^	0.5712	0.5724	0.5718	0.5794	0.6001	0.5898	0.5844	0.5971	0.5908	0.5729	0.5854	0.5791
PCDH^38^	0.6074	0.6155	0.6114	0.6273	0.6606	0.6439	0.5791	0.6195	0.5993	0.5997	0.5952	0.5974
CSQ^39^	0.5982	0.5756	0.5869	0.6302	0.6249	0.6275	0.6253	0.6208	0.6231	0.6430	0.6425	0.6428
DPN^40^	0.5400	0.5561	0.5480	0.5830	0.5534	0.5682	0.6307	0.6527	0.6417	0.6229	0.6366	0.6297
FAH^41^	0.5797	0.5943	0.5870	0.5808	0.6062	0.5935	0.5711	0.5642	0.5676	0.5798	0.5905	0.5851
DCPHA	**0.6600**	**0.6534**	**0.6567**	**0.6732**	**0.6912**	**0.6822**	**0.6700**	**0.6857**	**0.6779**	**0.6827**	**0.6798**	**0.6813**

Best Performance in Bold.

**Figure 3:**
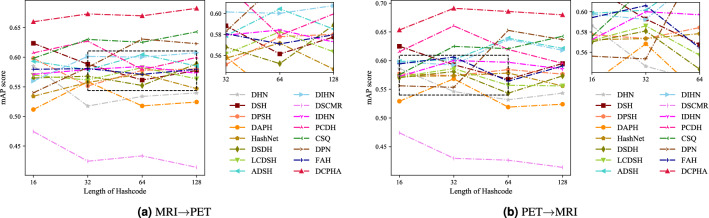
The precision curves of DCPHA and comparisons on ADNI2 dataset.

The results of neuroimage cross-modal retrieval on ADNI2 using mAP scores are shown in Table 2. As can be seen from the table, the proposed DCPHA outperforms 15 advanced counterparts. Regarding the average mAP score of 128 bits hash codes on ADNI2 dataset, DCPHA outperforms several sub-optimal models DIHN, DPN, and CSQ by $6.86\%$, $5.16\%$, and $3.85\%$ respectively. In other words, our method can significantly improve the performance of neuroimage cross-modal retrieval. For further comparison, the precision curve is plotted in Fig. 3. The experimental results are consistent with the retrieved mAP results in Table 2, where DCPHA has the best performance.

**Table 3:** The mAP scores of cross-modal retrieval on OASIS3 with different lengths of hash codes.

Method	16 bits			32 bits			64 bits			128 bits		
	M$\rightarrow$P	P$\rightarrow$M	Aver	M$\rightarrow$P	P$\rightarrow$M	Aver	M$\rightarrow$P	P$\rightarrow$M	Aver	M$\rightarrow$P	P$\rightarrow$M	Aver
DHN^27^	0.5982	0.5995	0.5989	0.5603	0.5676	0.5639	0.5947	0.5868	0.5908	0.5820	0.5850	0.5835
DSH^28^	0.6263	0.6262	0.6263	**0.6547**	**0.6681**	**0.6614**	0.6377	0.6279	0.6328	0.6337	0.6047	0.6192
DPSH^29^	0.6134	0.6123	0.6129	0.5877	0.6046	0.5962	0.5927	0.6013	0.5970	0.6016	0.6258	0.6137
DAPH^30^	0.5831	0.5913	0.5872	0.5604	0.5660	0.5632	0.5609	0.5652	0.5631	0.5705	0.5765	0.5735
HashNet^31^	0.6001	0.6110	0.6055	0.6304	0.6390	0.6347	0.5914	0.6227	0.6071	0.5969	0.6159	0.6064
DSDH^32^	0.5864	0.5978	0.5921	0.5979	0.6012	0.5996	0.6094	0.6248	0.6171	0.6333	0.6207	0.6270
LCDSH^33^	0.5880	0.6169	0.6024	0.5885	0.5988	0.5937	0.5793	0.5847	0.5820	0.5836	0.5904	0.5870
ADSH^34^	0.5835	0.5836	0.5836	0.5917	0.5922	0.5919	0.6182	0.6041	0.6111	0.6078	0.6152	0.6115
DIHN^35^	0.6124	0.6139	0.6131	0.6175	0.6377	0.6276	0.6068	0.6318	0.6193	**0.6343**	0.6345	0.6344
DSCMR^36^	0.5956	0.5905	0.5930	0.5756	0.5803	0.5780	0.5918	0.5953	0.5935	0.5856	0.5922	0.5889
IDHN^37^	0.6129	0.6005	0.6067	0.6035	0.6106	0.6071	0.6146	0.6189	0.6168	0.6209	0.6399	0.6304
PCDH^38^	0.5836	0.5568	0.5702	0.6062	0.6036	0.6049	0.6060	0.6186	0.6123	0.5919	0.6021	0.5970
CSQ^39^	0.6063	0.5997	0.6030	0.5942	0.5790	0.5866	0.6197	0.5921	0.6059	0.6016	0.6119	0.6068
DPN^40^	0.6151	0.5957	0.6054	0.6120	0.6091	0.6106	0.6007	0.5808	0.5907	0.6026	0.5916	0.5971
FAH^41^	0.5292	0.5335	0.5313	0.5328	0.5257	0.5292	0.5526	0.5478	0.5502	0.5899	0.5862	0.5881
DCPHA	**0.6491**	**0.6541**	**0.6516**	0.6495	0.6494	0.6495	**0.6633**	**0.6388**	**0.6511**	0.6335	**0.6487**	**0.6411**

Best Performance in Bold.

**Figure 4:**
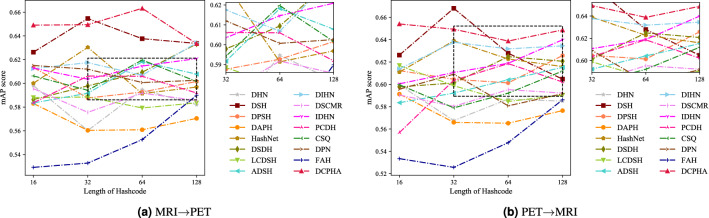
The precision curves of DCPHA and comparisons on OASIS3 dataset.

We evaluated DCPHA on OASIS3 dataset for cross-modal retrieval. The mAP scores of the retrieval are shown in Table 3. The experimental results show that DCPHA has the highest retrieval mAP scores in several metrics compared to 15 advanced retrieval methods. The proposed DCPHA improves $2.53\%$ over the best counterpart DSH from the average mAP score of 16 bits hash codes. Although the average mAP score of DSH with 32 bits hash codes is higher than DCPHA, the model constructed based on the multi-manifold property of data distribution has a great advantage in processing the multi-modal task method has a great advantage. The performance of proposed method is more stable on different lengths of hash codes. We plotted the precision curve to investigate the effectiveness of different methods for cross-modal retrieval on OASIS3 dataset as shown in Fig. 4. From the visualization, it is observed that the proposed DCPHA also outperforms all the compared methods, which is consistent with the retrieved mAP results.

### The further analysis of DCPHA

In this subsection, we will further analyze our proposed DCPHA from ablation experiments, hyper-parameters sensitivity analysis and comparison on natural image benchmark dataset.

#### Ablation experiments

The objective function of DCPHA is mainly composed of a multi-manifold similarity-preserving loss and a multi-semantic consistency loss. In order to research the contribution of these components to the model in more detail, we developed and evaluated two variants of DCPHA. i.e. DCHA and DPHA. DCHA only uses the multi-semantic consistency loss as the objective function and DPHA only uses the multi-manifold similarity-preserving loss as the optimization objective. Table 4 shows the results of ablation experiments on ADNI2. We found that both multi-manifold similarity-preserving and multi-semantic consistency contribute to the final retrieval performance of the model. DCHA obtains better performance when the hash codes is shorter, and the mAP score of DPHA is higher when the hash code is longer, which shows that optimizing the two objective functions at the same time is better than only optimizing one of them.

**Table 4:** The mAP of ablation experiments on ADNI2 with different lengths of hash codes.

Method	16 bits			32 bits			64 bits			128 bits		
	M$\rightarrow$P	P$\rightarrow$M	Aver	M$\rightarrow$P	P$\rightarrow$M	Aver	M$\rightarrow$P	P$\rightarrow$M	Aver	M$\rightarrow$P	P$\rightarrow$M	Aver
DCHA	0.6238	0.6672	0.6455	0.6345	0.6527	0.6436	0.6175	0.6173	0.6174	0.6083	0.6317	0.6200
DPHA	0.6274	0.6099	0.6186	0.6068	0.6482	0.6275	0.6361	0.6313	0.6337	0.6387	0.6322	0.6355

#### Hyper-parameter sensitivity analysis

**Figure 5:**
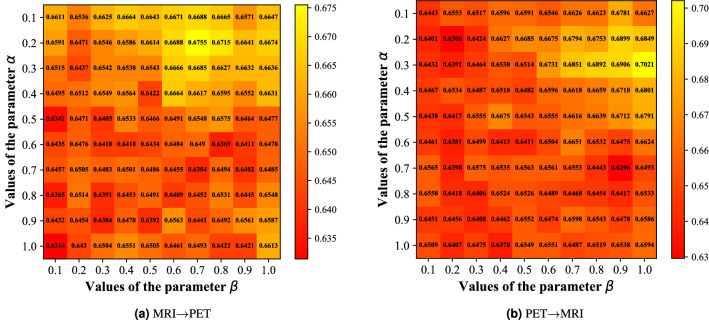
The mAP scores on ADNI2 with hyper-parameters in the range of [0.1, 1].

The objective function of DCPHA contains two hyper-parameters $\alpha$ and $\beta$, and we investigate the effect of the hyper-parameters that control the weight ratio between the losses in Eq. (10). First, we fix the length of the hash code *mathcal* *K* to 32. Then, we keep $\alpha$ and $\beta$ in the range of [0.1, 1] to calculate the MAP score. The result is shown in the Fig. 5. It is clear that different hyperparameters yield different performance. Considering from the average MAP, we finally chose $\alpha =0.3$ and $\beta =1$ as hyper-parameters for the ADNI2 dataset. By using the same scheme, we can obtain the optimal values of hyper-parameters for $\alpha =0.1$ and $\beta =1.0$ on the OASIS3 dataset.

#### Experiments on natural image benchmark datasets

In order to further measure the fitting and generalization ability of DCPHA, we conduct comparative experiments with 10 advanced cross-modal retrieval methods on the natural image benchmark datasets MIRFLICKR25K. In our experiments, we follow the dataset partition and feature exaction strategies from^36, 42^. In this experiment, we report the mAP scores of the compared methods for two different cross-modal retrieval tasks: 1) retrieving text using image queries (I$\rightarrow$T) and 2) retrieving images using text queries (T$\rightarrow$I). The experimental results obtained in “I$\rightarrow$T” and “T$\rightarrow$I” tasks on MIRFLICKR25K are shown in Table 5. Since our proposed multi-semantic consistency and multi-manifold similarity preserving constraints based on the multi-manifold property of multi-modal hash codes, DCPHA achieves a significant performance improvement on the multi-label benchmark dataset, i.e., MRIFLICKER25K.

**Table 5:** The mAP scores of cross-modal retrieval on MIRFLICKER25K with different lengths of hash codes.

Method	16 bits			32 bits			64 bits			128 bits		
	I$\rightarrow$T	T$\rightarrow$I	Aver	I$\rightarrow$T	T$\rightarrow$I	Aver	I$\rightarrow$T	T$\rightarrow$I	Aver	I$\rightarrow$T	T$\rightarrow$I	Aver
DSH^28^	0.6284	0.6378	0.6331	0.6244	0.6271	0.6257	0.6009	0.6122	0.6066	0.5776	0.5626	0.5701
DPSH^29^	**0.6993**	0.6971	0.6982	0.7011	0.7016	0.7014	0.7020	0.6992	0.7006	0.7025	0.6996	0.7010
LCDSH^33^	0.6806	0.6941	0.6873	0.6828	0.6919	0.6873	0.6851	0.6967	0.6909	0.6893	0.6940	0.6916
ADSH^34^	0.6891	0.6939	0.6915	0.6905	0.6936	0.6920	0.6901	0.6935	0.6918	0.6910	0.6941	0.6925
DSCMR^36^	0.6513	0.6671	0.6592	0.6748	0.6891	0.6820	0.6849	0.6883	0.6866	0.6868	0.6895	0.6881
IDHN^37^	0.6663	0.6608	0.6635	0.6518	0.6393	0.6456	0.6401	0.6311	0.6356	0.6344	0.6241	0.6293
DBDH^43^	0.6974	0.6973	0.6973	0.7006	0.6972	0.6989	0.7006	0.6971	0.6988	0.7008	0.6987	0.6997
PCDH^38^	0.6460	0.6407	0.6433	0.6350	0.6236	0.6293	0.6102	0.6293	0.6197	0.6171	0.6026	0.6099
DPN^40^	0.6790	0.6749	0.6770	0.6493	0.6692	0.6592	0.6905	0.6808	0.6857	0.6906	0.6916	0.6911
QSMIH^44^	0.6619	0.6615	0.6617	0.6687	0.6616	0.6652	0.6740	0.6693	0.6716	0.6805	0.6700	0.6752
DCPHA	0.6989	**0.7046**	**0.7017**	**0.7045**	**0.7053**	**0.7049**	**0.7060**	**0.7065**	**0.7062**	**0.7054**	**0.7066**	**0.7060**

Best Performance in Bold.

## Conclusion and future work

In this paper, we proposed a deep consistency-preserving hash auto-encoders model, called DCPHA, based on the multi-manifold property of hash codes distributed in Hamming space to solve the problem of lack of discriminability of hash codes with the same semantics. Specifically, DCPHA consists of a pair of asymmetric auto-encoders and two semantics-preserving attention branches that work in the feature encoding stage and hash decoding stage, respectively. In addition, two constraints, namely multi-semantic consistency and multi-manifold similarity-preserving, were embedded in the learning of hash codes. We theoretically demonstrated that our proposed multi-manifold similarity-preserving has manifold preserving invariance. As the experimental results show, the proposed DCPHA can obtain state-of-the-art performance on simple medical multi-modal image datasets (i.e., ADNI2) and multi-label natural image datasets (i.e., MIRFLICKER25K). In future work, we will build a medical multi-modal database, including diagnostic reports, audio, and construct a multi-modal hash method to accomplish mutual retrieval of data from multiple sources. And we will further explore the impact of multi-view on the generation of hash codes for multi-modal samples.

## Supplementary Information

Supplementary Information.

## Data Availability

The datasets generated during and analysed during the current study are available from the corresponding author on reasonable request.
