# Supplementary material for: Deep consistency-preserving hash auto-encoders for neuroimage cross-modal retrieval
Source: Sci Rep. 2023 Feb 9;13:2316. doi: 10.1038/s41598-023-29320-6 (PMC9911775; doi:10.1038/s41598-023-29320-6)
Supplement: Supplementary file 1 — Supplementary Information. [file 41598_2023_29320_MOESM1_ESM.pdf]

Mathematical proof of Theorem 1

Laplacian Eigenmaps use the correspondence between the Laplace and the Laplace-Beltrami operator on manifold, and the connections to heat equation, and proposed a non-linear dimensionality reduction method from Riemann space to Euclidean space. The function as follows Eq. (1):

$$\mathscr{L}_{laplacican} = \sum_{m,n=1}^{M} \sum_{i,j=1}^{N} \frac{1}{2} S_{i,j} \times \parallel H_i^m - H_j^n \parallel_2^2 \tag{1}$$

Proof : Expand Eq. (1) to Eq. (2):

$$\mathscr{L}_{laplacican} = \sum_{m,n=1}^{M} \sum_{i,j=1}^{N} \frac{1}{2} S_{i,j} \times \left(\parallel H_i^m \parallel_2^2 + \parallel H_j^n \parallel_2^2 + 2 \left(H_i^m\right)^T H_j^n \right) \tag{2}$$

where $\parallel H_i^m \parallel_2^2 + \parallel H_j^n \parallel_2^2 = 2$, and $\sum_{m,n=1}^{M} \sum_{i,j=1}^{N} S_{i,j}$ is a constant defined as T, Eq. (2) can be written to Eq. (3):

$$\mathscr{L}_{laplacican} = T - \sum_{m,n=1}^{M} \sum_{i,j=1}^{N} S_{i,j} \times \left(H_i^m\right)^T H_j^n \tag{3}$$

Expanding $\log\left(1 + e^{S_M\left(h_i^m, h_j^n\right)}\right)$ according to the power series expansion principle, $\mathscr{J}_2$ can be written as Eq. (4).

$$\begin{aligned} \mathscr{J}_2 = & \sum_{m,n=1}^{M} \sum_{i,j=1}^{N} \sum_{u=2}^{+\infty} \left((-1)^{u-1} \times \frac{\left(e^{S_M\left(h_i^m, h_j^n\right)}\right)^u}{u} \right) \\ & + e^{S_M\left(h_i^m, h_j^n\right)} - I\left(\ell_i, \ell_j\right) \times S_M\left(h_i^m, h_j^n\right) \end{aligned} \tag{4}$$

Then expanding $e^{S_M\left(h_i^m,\, h_j^n\right)}$ by the *Taylor expansion principle*, Eq. (4) can be written as Eq. (5).

$$\begin{aligned} \mathscr{J}_2 = & \sum_{m,n=1}^{M} \sum_{i,j=1}^{N} \sum_{u=2}^{+\infty} \left((-1)^{u-1} \times \frac{\left(e^{S_M\left(h_i^m,\, h_j^n\right)}\right)^u}{u} \right) \\ & + \sum_{u=0}^{+\infty} \left(\frac{S_M\left(h_i^m,\, h_j^n\right)^u}{u!} \right) - I\left(\ell_i,\, \ell_j\right) \times S_M\left(h_i^m,\, h_j^n\right) \end{aligned} \tag{5}$$

where $\sum_{u=0}^{+\infty} \left(\frac{S_M\left(h_i^m,\, h_j^n\right)^u}{u!} \right) = 1 + S_M\left(h_i^m,\, h_j^n\right) + \sum_{u=2}^{+\infty} \left(\frac{S_M\left(h_i^m,\, h_j^n\right)^u}{u!} \right)$, Eq. (5) is written as Eq. (6).

$$\begin{aligned} \mathscr{J}_2 = & \sum_{m,n=1}^{M} \sum_{i,j=1}^{N} \sum_{u=2}^{+\infty} \left((-1)^{u-1} \times \frac{\left(e^{S_M\left(h_i^m, h_j^n\right)}\right)^u}{u} + \frac{S_M\left(h_i^m, h_j^n\right)^u}{u!} + 1 \right) \\ & + S_M\left(h_i^m, h_j^n\right) - I\left(\ell_i, \ell_j\right) \times S_M\left(h_i^m, h_j^n\right) \end{aligned} \tag{6}$$

Further disaggregation of Eq. (6) to Eq. (7).

$$\mathscr{J}_2 = \sum_{m,n=1}^{M}\sum_{i,j=1}^{N}\sum_{u=2}^{+\infty}\left((-1)^{u-1}\times\frac{\left(e^{S_M\left(h_i^m,\ h_j^n\right)}\right)^u}{u}+\frac{S_M\left(h_i^m,\ h_j^n\right)^u}{u!}+1\right)$$
$$+\sum_{m,n=1}^{M}\sum_{i,j=1}^{N}\left(\left(1-I\left(\ell_i,\ \ell_j\right)\right)\times S_M\left(h_i^m,\ h_j^n\right)\right) \tag{7}$$

Since Laplacian Eigenmaps directly uses labels to construct the similarity matrix S, $1-I\left(\ell_i,\ell_j\right)=-S_{i,j}$, the above equation can be written as Eq. (8):

$$\mathscr{J}_2 = \sum_{m,n=1}^{M}\sum_{i,j=1}^{N}\sum_{u=2}^{+\infty}\left((-1)^{u-1}\times\frac{\left(e^{S_M\left(h_i^m,\ h_j^n\right)}\right)^u}{u}+\frac{S_M\left(h_i^m,\ h_j^n\right)^u}{u!}+1\right)$$
$$-\sum_{m,n=1}^{M}\sum_{i,j=1}^{N}\left(S_{i,j}\times S_M\left(h_i^m,h_j^n\right)\right) \tag{8}$$

We have used the cosine metric to construct the homogeneous manifold similarity. When $m=n, S_M\left(h_i^m,\ h_j^n\right)=\frac{h_i^{\cdot T}\cdot h_j^{\cdot}}{||h_i^{\cdot}||\cdot||h_j^{\cdot}||}=\left(H_i^m\right)^T H_j^n$. The above equation can be rewritten as Eq. (9):

$$\mathscr{J}_2 = \sum_{m,n=1}^{M}\sum_{i,j=1}^{N}\left(log\left(1+e^{S_M\left(h_i^m,h_j^n\right)}\right)-S_M\left(h_i^m,h_j^n\right)\right)$$
$$-\sum_{m,n=1}^{M}\sum_{i,j=1}^{N}\left(S_{i,j}\times\left(H_i^m\right)^T H_j^n\right) \tag{9}$$

Since subject to $log\left(1+e^{S_M\left(h_i^m,h_j^n\right)}\right)=2S_M\left(h_i^m,h_j^n\right)$, therefore $\sum_{m,n=1}^{M}\sum_{i,j=1}^{N}\left(log\left(1+e^{S_M\left(h_i^m,h_j^n\right)}\right)-S_M\left(h_i^m,h_j^n\right)\right)=\sum_{m,n=1}^{M}\sum_{i,j=1}^{N}S_M\left(h_i^m,h_j^n\right)=T$, then the Eq. (9) is equivalent to Eq. (10).

$$\mathscr{J}_2 = T-\sum_{m,n=1}^{M}\sum_{i,j=1}^{N}\left(S_{i,j}\times\left(H_i^m\right)^T H_j^n\right) \tag{10}$$

After the above derivation, combining Eq. (3) and Eq. (10), muliti-manifold similarity-preserving loss $\mathscr{J}_2$ is equivalent to Laplacain Eigenmaps, *i.e.* $\mathscr{J}_2$ has the manifold preserving invariance.
End Proof.
